# Exploiting *Citrus aurantium* seeds and their secondary metabolites in the management of Alzheimer disease

**DOI:** 10.1016/j.toxrep.2020.06.001

**Published:** 2020-06-05

**Authors:** Doha H. Abou Baker, Bassant M.M. Ibrahim, Nabila S. Hassan, A.F Yousuf, Souad El Gengaihi

**Affiliations:** a Medicinal and Aromatic Plants Department. National Research Centre, Dokki, Giza. PO 12622, Egypt; b Pharmacology Department. Medical Research Division. National Research Centre, Dokki, Giza, PO 12622, Egypt; c Pathology Department. Medical Research Division. National Research Centre, Cairo, PO 12622, Egypt; d Physiology Department. Faculty of Medicine for Girls, Al-Azhar University, Egypt

**Keywords:** *Citrus aurantium* seeds, Flavonoids, Lemonoids, Alzheimer disease, Acetylcholine esterase, Tau protein, Beta-Amyloid protein, Y-Maze, Open field

## Abstract

Fruit by-products are considered nature’s golden gift for human health and a good starting point to discover new drugs depending on the fact that they contain millions of bio-active compounds that are responsible for therapeutic activities. In this context, the main goal of this study is to recycle *Citrus aurantium* (*C. aurantium*) seeds to produce pharmaceutical molecules to be used in the prevention of the progressive neurological damage associated with Alzheimer disease (AD). Donepezil (0.75 mg/kg), hesperidin (125 and 250 mg/kg) and limonoids (50 and 100 mg/kg) were used for treatment of rats for 2 weeks prior to concomitant administration of AlCl_3_ for three successive weeks. Protection against cognitive deterioration was observed among study group with insignificant difference from normal control group and significant difference from positive control group in the Y-Maze test. On the other hand, treatment with both doses of hesperidin (125 and 250 mg/kg) and high dose of limonoids only (100 mg/kg) produced improvement in psychological state, observed by significant increase in ambulation frequency in comparison to positive control group, however it was not as frequent as normal group, as it was significantly less than normal group in the open field test. Regarding acetylcholine esterase (AChE) and beta-amyloid (β amyloid) levels, the effect of limonoids low dose was the best as it didn’t have a significant effect when compared to normal control, also hesperidin in both doses showed insignificant effects on β amyloid levels when compared to normal control group. Our results encourage the use of *C. aurantium* seeds which are wasted in huge amounts, as Alzheimer prophylactic food additives.

## Graphical abstract

**Figure:**
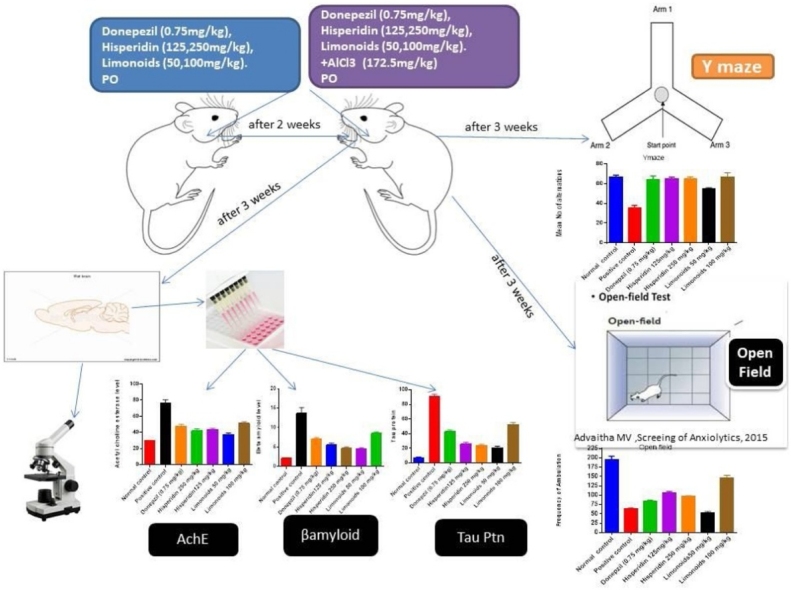

## 1 Introduction

Alzheimer's disease (AD) treatment is very expensive lying beyond affordable limits of low to middle-income individuals in most societies. Manifestations of AD include progressive amnesia that starts first with forgetting recent events while old memories are preserved, followed by more sophisticated disabilities like losing the ability to calculate and to use common objects and tools due to degeneration of hippocampus and cortical neurons [1].

There are lots of environmental risk factors for AD which has not been certainly identified. Factors that play a role in AD development include accidental or intentional exposure to metals as aluminum or silica that are present in the soil, water and cooking pots [2]. Diseases that increase the risk of incidence of AD are strokes, inflammation and oxidative stress in addition to alcoholism and cigarette smoking [3].

Treatment of AD includes donepezil which is an AChE inhibitor that acts on the central nervous system, but it has not been shown to change the progression of the disease. That is why treatment should be stopped if no benefit is seen. It also exhibits side effects as nausea, disturbed sleeping, agitation, diarrhoea, lethargy, and moreover dangerous side effects like abnormal heart rhythms, difficulty in emptying urine from the bladder, and seizures" [4].

Such undesirable effects represented the inspiring motive to use new natural herbal products that have proven efficacy against cerebrovascular diseases by acting in different mechanisms. However, their use is still limited by deficient information regarding their toxicity or efficacy when compared with standard medications, beside the problem of deficient ingredient standardization of their ingredients [5]. Yet these problems can be overcome by vigorous recent researches for standardization, as well as pharmacological experimental studies for the detection of toxicity and efficacy of plant by-products. Recently there is an increase in the use of plant by-products depending on their availability, bioavailability, potentiality, safety and low cost in comparison to modern therapeutic drugs for the treatment of dangerous diseases [6]. Fruit by-products could be regarded as precious source of polyphenols; a natural antioxidant. Polyphenol is used for the management of AD and cancer diseases [[7], [8], [9], [10]]. Hesperidin is a biologically active flavonoid found in Citrus with good anti-oxidative, antihypertensive, anti-hyperlipidemia, anti-diabetic, anti-inflammatory, and hepato-protective potentials [[11], [12], [13], [14]].

Citrus fruits represent the biggest fruit sector production all over the world, and their peels act as the dominant by-product of *C. aurantium* processing industries [15]. These *C. aurantium* fruit residues, which are generally discarded as waste in the environment, can act as potential nutraceutical resources. Due to their low cost and availability, such wastes are capable of offering significant low-cost nutritional dietary supplements. The utilization of these bioactive rich *C. aurantium* residues can provide an efficient, inexpensive, and environment-friendly platform for the production of novel nutraceuticals or for the improvement of older ones.

*C. aurantium* seeds contain limonoids and ﬂavonoids as their major bioactive constituents. The most abundant *C. aurantium* flavonoids, generally known as the flavanones, include hesperidin, naringin, narirutin, and neohesperidin. Such compounds have been found to provide health benefits due to their antioxidative, anticancer, anti-inflammatory, and cardiovascular protective activities. Furthermore, the consumption of naringin and hesperidin reduce cholesterol levels in hamsters by 32–40% [16].

Limonoids are a unique class of highly oxygenated tetracyclic triterpenoids, Members of the class limonoids have wide health-promoting and disease-preventing activities, including anticancer, antibacterial, antioxidant, larvicidal, antimalarial and antiviral activities, and thus they possess potential applications in nutraceuticals, pharmaceuticals, and agriculture [17].

Herein, our study promoted the use of *C. aurantium* seeds, which are abundant cheap natural products disposed as waste in huge amounts, as protective agent against behavioural deterioration as well as biochemical and histopathologic changes in brains of rats, mimicking AD which is induced by the use of AlCl_3_.

## 2 Material and methods

### 2.1 Plant material

*C. aurantium* fruits were purchased from the local market of Dokki, Egypt. The identification of *C. aurantium* was confirmed by Dr. Mona M. Marzouk, Department of Phytochemistry and Plant Chemosystematics, National Research Center (NRC), Cairo, Egypt. The *C. aurantium* fruits seeds were separated from fruits; air dried then ground to a fine powder. Grinding was necessary to improve extraction efficiency.

### 2.2 Preparation of crude limonoids

Fifty grams of powdered seeds were placed in a Soxhlet apparatus and washed overnight with hexane to remove the oil, then extracted with acetone (IL X3 times). After removal of the solvent under reduced pressure, the crude extract (8.0 g) was suspended in H_2_O (1.5 L) and partitioned with CH_2_C1_2_ and isopropanol used in a ratio of 4 to 1 to isolate crude limonoid aglycones according to Qin et al. [18]. Crude limonoids were tested with p-dimethyl amino benzaldehyde reagent (Ehrlich's reagent). Crude lemonoids were analysed by TLC silica gel F254 and eluted with ethyl ether-acetic acid-water (15:3:1) giving ten spots. Their Rf values were then compared to those found in published literature [19] and authentic compounds values.

### 2.3 Preparation of crude hesperidin

Air-dried seeds were ground into powder and kept in Soxhlet with petroleum ether as a solvent. After the *C. aurantium* fruit seeds have been completely defatted, the material remaining in the flask was soaked with methanol and the methanolic extract was concentrated under the reduced pressure then the residue was washed with aqueous acetic acid (6%) to precipitate hesperidin. Crude hesperidin gave red color with ferric chloride test whereas it gave violet color on Shinoda test. Two spots were observed in thin layer chromatography of crude hesperidin using n-Butanol: Acetic Acid: Water (3:1:1) as mobile phase at 0.20 and 0.62 Rf according to published literature [20]. The flavonoid glycoside, hesperidin, colourless needles were separated and used for the investigation.

### 2.4 Pharmacological study

#### 2.4.1 Materials

##### 2.4.1.1 Animals

Female Wistar Albino rats, weighing 180–200 g were used. The rats were obtained from the animal house colony of the National Research Centre (NRC), Egypt. The animals were kept in standard plastic cages in an air-conditioned room at 22 ± 3 °C, 55 ± 5 % humidity and supplied with standard laboratory diet and water ad libitum. All experimental procedures were conducted in accordance with the guide for the care and use of laboratory animals and and the animal procedures were performed in accordance with the Ethics Committee of the National Research Centre with approval certificate registration number 16/138, Experimental procedures and use of laboratory animals followed the recommendations of the National Institutes of Health (Publication No. 85-23, revised 1985).

##### 2.4.1.2 Drugs and tested compounds

Donepezil hydrochloride, (RS)-2-[(1-benzyl-4-piperidyl) methyl]-5,6-dimethoxy-2,3-dihydroinden-1-one (Aricept; Pfizer Inc, New York, NY, USA), was administered daily by oral gavage (p.o) at a dosage of 0.75 mg/kg bw. The donepezil dosage was chosen on the basis of information from previous *in vivo* studies [21]. Hesperidin was given p.o in two doses (125 and 250 mg/kg) that were selected according to acute toxicity study. Limonoid was given p.o in two doses (50 and 100 mg/kg) that were selected from literature [22]. All tested agents were given dissolved in 2 mL of distilled water.

##### 2.4.1.3 Behaviour stress test apparatuses

Locally made wooden Y maze and open field in the carpentry were used.

#### 2.4.2 Methods

##### 2.4.2.1 Acute toxicity study

Healthy young adult female Wister albino rats aged 12 weeks and weighed from 180 to 200 g were used in the experiment. Care was taken that the rats weren’t pregnant. The animals were kept for five days before the test under housing and feeding conditions mentioned before. Animals were kept fasting overnight, then weighed and the dose of aqueous extract of hesperidin that would be given to each rat was calculated according to body weight, Aqueous extract of hesperidin was prepared just before administration orally to five female rats in doses of 2.5 gm/kg, the rat weighing 200 g was given 500 mg, while a rat weighing 180 g was given 450 mg of hesperidin dissolved in 2 mL distilled water. Another five female rats served as negative controls and were given 2 mL of distilled water. Animals were observed individually once during the first 30 min after dosing, then periodically during the first 24 h (and special attention was given during the first 4 h). After that the animals were kept under observation for any change in behaviour, bowel habits, marked weight loss or mortality for the following 14 days after administration of hesperidin. Acute toxicity study followed the OECD test guideline 425 (2008).

There were no mortalities recorded, or signs of toxicity, in the next 14 days, hence acute toxicity study revealed that extract of hesperidin is non-toxic up to 2.5 g/kg b.w. Accordingly the selected doses of hesperidin were used for the efficacy study as 1/10 (250 mg/kg) and 1/20 (125 mg/kg) of the tested dose for the acute toxicity study. The rat weighing 200 g was given (50 and 25) mg of hesperidin dissolved in 2 mL distilled water.

##### 2.4.2.2 Induction of AD

Four selected doses of Aluminium chloride (AlCl_3_) (17, 50, 100 and 172.5 mg/kg) -as reported by previous studies- were used to choose the best dose which causes the fastest deterioration in cognition with the least mortality [[23], [24], [25], [26]]. All doses were dissolved in distilled water and given orally daily to four groups of female rats (each consisted of ten rats). Each rat received a fixed volume of AlCl_3_ solution (2 mL). A fifth group served as a negative control and was given distilled water (2 mL). Cognitive assessment was done weekly, by using the Y-Maze test. The dose which produced the most cognitive dysfunction in rats after three weeks of admission was 172.5 mg/kg without any mortality.

##### 2.4.2.3 Protective efficacy study

###### 2.4.2.3.1 Study design

Seventy adult female Wistar Albino rats were enrolled in this study and were divided into 7 groups (ten each). They were classified as follows: First group: Negative control group in which rats were given a daily oral dose of 2 mL distilled water throughout the experiment. Second group: Positive control in which induction of animal model mimicking AD was done by daily oral administration of AlCl_3_ to rats in a dose of 172 mg/kg for three successive weeks [24].Third to seventh groups: Treated rats were orally given donepezil hydrochloride (0.75 mg/kg) used as a standard drug, hesperidin (125, 250 mg/kg) and limonoids (50,100 mg/kg), respectively, for two weeks followed by the combination of each treatment with AlCl_3_ for another three successive weeks.

###### 2.4.2.3.2 Assessment of the protective effects of hesperidin and limonoids

At the end of the experimental period (after five weeks) and 24 h after the last dose behaviour stress tests were done.

###### 2.4.2.3.3 Behaviour stress tests

####### Spontaneous alternation Y-Maze

*Principle:* Continuous Spontaneous Alteration behaviour was examined using the Y maze apparatus. It is well known that Spontaneous Alternation is a measure of spatial working memory. The Y maze can be used as a measure of short term memory. Y Maze Spontaneous Alternation is a behavioral test for measuring the willingness of rodents to explore new environments. Rodents typically prefer to investigate a new arm of the maze rather than returning to one that was previously visited.

**Fig. 1:**
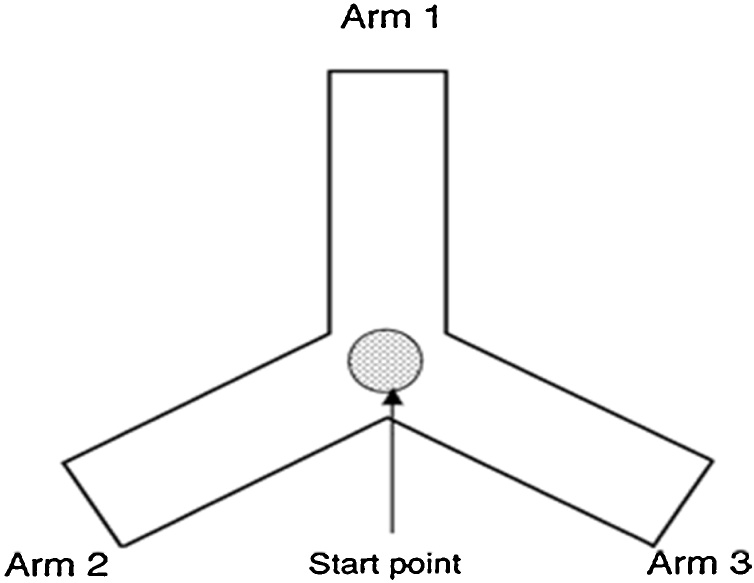
Structure of spontaneous alternation of Y maze apparatus.

*Procedure* : Spontaneous Alternation was assessed using a Y-Maze (Fig. 1) composed of three equally spaced arms (32 cm long, 10 cm high and 5 cm wide) extending from a central platform at 120°(labelled A, B, and C). The test was performed according to previously published protocols. Each rat was placed at the centre of the maze as shown in the figure and allowed to move freely through the maze during a session lasting 8 min. The Arm entry was defined as the entry of 4 paws into one arm. The sequence of arm entries was recorded visually, between each session the maze was cleaned with 70 % ethanol and left to dry completely to prevent olfactory cues. Alternation was defined as multiple entries into the 3 arms (A, B or C) on overlapping triplet sets, for instance, if the animal makes the following arm entries; ACBCABCACABCA, in this example, the animal made 13 ar m entries 8 of which were correct alternations [26].

The percentage of Spontaneous Alternation was calculated as the ratio of actual to possible alternations (defined as the total number of arm entries minus 2), multiplied by 100 as shown in the following equation: %Alternation = [number of alternations/(number of choices-2)]x100.

####### Open field test

*Principle* : The open field test aims at evaluating the psychological state of animals.

*Procedure* : The open field test was carried out in a square wooden arena (80 cm · 80 cm · 40 cm high) with red walls and a white smooth polished floor divided by black lines into 16 equal squares. The test was performed under white light in a quiet room. Each rat was placed at the same corner square and observed for 5 min. The floor and walls were cleaned after testing each rat. The ambulation frequency: the number of squares crossed by the animal was recorded during the 5 min observation period. Rearing frequency: number of times the animal stood stretched on its hind limbs with or without forelimb support [27].

##### 2.4.2.4 Brain tissue sampling and preparation

###### 2.4.2.4.1 Biochemical parameters

At the end of the experimental period (after five weeks), the animals were kept fasting for 12 h and killed by decapitation. The whole-brain of each animal was rapidly dissected, thoroughly washed with isotonic saline, dried, and then weighed. Thereafter, each brain was sagitally divided into two portions. The first portion of each brain was homogenized immediately to give a 10 % (w/v) homogenate in ice-cold medium containing 50 mmol/l Tris-HCl (pH 7.4) and 300 mmol/l sucrose. The homogenate was centrifuged at 3000 rpm for 10 min at 41 °C. The supernatant (10 %) was separated for biochemical analysis (AChE, tau proteins and beta- amyloids) using ELlSA Kits.

###### 2.4.2.4.2 Histopathological examination

The second portion of each brain was fixed in formalin buffer (10 %) for 24 h. The brains were washed in tap water and then dehydrated using serial dilutions of alcohol (methyl, ethyl, and absolute ethyl). Specimens were cleared in xylene and embedded in paraffin in a hot air oven at 561C for 24 h. Paraffin beeswax blocks were prepared for sectioning at 4 mm using a microtome. The obtained tissue sections were collected on glass slides, deparaffinised, and stained with hematoxylin and eosin stains, for histopathological examination using a light microscope [28].

## 3 Statistical analysis

The comparison between means was carried out using one-way analysis of variance (ANOVA) followed by the Tukey Kramer multiple comparison test. P < 0.05 was considered as being significant in all types of statistical tests. Graph pad prism software (version 6) was used to carry out all statistical tests.

## 4 Results

### 4.1 Behaviour stress tests

#### 4.1.1 The Y- maze test results

The present study revealed that oral administration of AlCl_3_ in a dose of 172 mg/kg for three successive weeks caused marked deterioration in cognition, observed by a significant reduction in percentage of alternation of rats in the Y Maze test.

Treatment of rats with donepezil used as a standard drug (0.75 mg/kg), hesperidin (125 and 250 mg/kg) and limonoids (50 and 100 mg/kg) for 2 weeks prior to concomitant administration of AlCl_3_ for three successive weeks produced protection against cognitive deterioration, observed by insignificant difference from normal control group and significant difference from positive control group. There was no significant difference observed for alternation between all treated groups.

The percentage of alternation was calculated between groups in relation to normal control and positive control groups. It was found that the lowest percentage of alternation in relation to normal group was that of positive control followed by the low dose of limonoids, while the highest percentage of alternation in relation to normal approximating the normal level was that of limonoids high dose followed by hesperidin high dose then low dose then donepezil, respectively. Results are expressed in Table 1.

**Table 1:** Effect of hesperidin (125 and 250 mg/kg) and limonoids (50 and 100 mg/kg) on cognitive functions of AD induced rats tested by Y-Maze test.

Group	Negative control (2 mL distilled water)	Positive control AlCl_3_ (172 mg/kg)	Donepezil (0.75 mg/kg)	Hesperidin (125 mg/kg)	Hesperidin (250 mg/kg)	Limonoids (50 mg/kg)	Limonoids (100 mg/kg)
Y-Maze Test							
Mean number of alternation +SE	66.78+1.95	35.38+2.6^@^	63.79+4.07*	64.5+2.03*	64.79+2.04*	55.03+1.13*	66.24+4.89*
% of alternation from negative control group	----------	52.98	95.52	96.58	97.02	82.4	99.19
% from positive control group (AlCl_3_172 mg/kg)	188.75	----------	180.29	182.3	183.12	155.53	187.22

Results are expressed as means of % of alternations ± SE, n = 10. Significance at p < 0.05 was measured for % of alternation in the Y maze arms.@ Significantly different from negative control group.*Significantly different from positive control group.

#### 4.1.2 The Open Field test results

The present study revealed that oral administration of AlCl_3_ for three successive weeks caused marked deterioration in the psychological state, observed by a significant reduction in the number of movements (ambulation frequency) of rats in the open-field arena.

Treatment of rats with donepezil used as a standard drug (0.75 mg/kg) and limonoids (50 mg/Kg) concomitant with AlCl_3_ didn’t improve the psychological state, observed by the insignificant change from the positive control group. On the other hand treatment with both doses of hesperidin (125 and 250 mg/kg) and the high dose of limonoids (100 mg/kg) produced improvement in psychological state, observed by a significant increase in ambulation frequency in comparison to the positive control group, however, it was not as frequent as a normal group, as it was significantly less than normal group.

The percentage of ambulation was calculated between groups in relation to normal control group. It was found that the lowest percentage of ambulation in relation to normal group was that of the low dose of limonoids followed by positive control, while the highest percentage of ambulation in relation to normal level was that of limonoids high dose followed by hesperidin low dose then high dose then donepezil, respectively.

Regarding rearing which reflects tendency to discover surroundings, it was found that groups treated with both doses of hesperidin and low dose of limonoids weren’t significantly better than positive control group, but the high dose limonoids group, as well as the donepezil group, were significantly better than the positive control group yet they were significantly worse than the normal control group.

Also the percentage of rearing was calculated between groups in relation to normal control group. It was found that the lowest percentage of rearing in relation to normal group was that of the low dose of limonoids followed by hesperidin low dose, hesperidin high dose then positive control respectively, while the highest percentage of rearing in relation to normal level was that of limonoids high dose followed by donepezil.Results are expressed in Table 2.

**Table 2:** Effect of hesperidin (125 and 250 mg/kg) and limonoids (50 and100 mg/kg) on psychological state of AD induced rats tested by open field test.

Group		Negative control (2 mL distilled water)	Positive control AlCl_3_ (172 mg/kg)	Donepezil (0.75 mg/kg)	Hesperidin (125 mg/kg)	Hesperidin (250 mg/kg)	Limonoids (50 mg/kg)	Limonoids (100 mg/kg)
Open Field Test								
Ambulation	Frequency	195+9.57	62.5+3.22^@^	83.33+3.33^@^	106.7+3.33^@^*^#&^	96.67+1.66^@^*^#&^	53.33+3.33^@$&^	143.3+6.66^@^*^$^
	% fromnegative control	-------	32.05	42.73	54.71	49.57	27.34	73.48
Rearing	Frequency	17.75+0.85	5.5+0.64^@^	8.5+0.28^@^*	4.33+0.33^@$&^	5.33+0.33^@$&^	4+0.57^@$^^&^	8.66+0.33^@^*
	% from negative control	-------	30.98	47.88	24.39	30.02	22.53	48.78

Results are expressed as means ± SE, n = 10. significance at p < 0.05.@ Significantly different from normal control, *Significantly different from positive control group.$ Significantly different from donepezil (0.75 mg/kg) group. # Significantly different from limonoids (50 mg/kg) group& Significantly different from limonoids (100 mg/kg) group.

### 4.2 Biochemical parameters

The present study revealed that oral administration of AlCl_3_ for three successive weeks caused significant increase in AChE, tau protein and beta-amyloid, which are markers of AD, when compared to normal control and all treated groups.

Treatment of rats with donepezil used as a standard drug (0.75 mg/kg), hesperidin (125 and 250 mg/kg) and limonoids (50 and 100 mg/kg) for 2 weeks prior to concomitant administration of AlCl_3_ for three successive weeks produced significant reduction in AChE, tau protein and beta-amyloid, when compared to positive control group that received AlCl_3_. However, the increase in tau protein was also significant for all groups when compared to normal control group. Regarding Ach-E and beta-amyloid levels, the effect of limonoids low dose was the best as it didn’t have a significant effect when compared to normal control, also hesperidin in both doses showed insignificant effects on beta-amyloid levels when compared to normal control group. The results are expressed in Table 3.

**Table 3:** Effect of hesperidin (125and250 mg/kg) and limonoids (50 and 100 mg/kg) AChE, Tau protein and β amyloid in brain homogenates of AD induced rats.

Group	Negative control (2 mL distilled water)	Positive control AlCl_3_ (172 mg/kg)	Donepezil (0.75 mg/kg)	Hesperidin (125 mg/kg)	Hesperidin (250 mg/kg)	Limonoids (50 mg/kg)	Limonoids (100 mg/kg)
BiochemicalParameter							
Ach E (μ/mg protein)	29.84+2.22	76.13+4.29^@^	47.7+2.36^@^*	43.65+1.39^@^*	42.18+2.17^@^*	37+2.23*^&^	51.28+2.02^@^*
Tau protein (pg/mg protein)	7.4+0.74	90.6+ 3.13^@^	42.85+2.1^@^*	25.93+2.47^@*$&^	24.28+1.43^@*$&^	20.87+2.24^@*^^$^	52.8+2.37^@^*
Beta-amyloid (ng/mg protein)	2.14+0.08	13.7+1.46^@^	7.05+0.43^@^*	5.57+0.45*	4.65+0.4*^&^	4.59+0.25*^&^	8.52+0.36^@^*

Results are expressed as means of levels of AcH-E, Tau protein and βamyloid in brain tissue homogenates of rats ± SE, n = 10. significance at p < 0.05.@Significantly different from normal control *Significantly different from positive control.$ Significantly different from donepezil (0.75 mg/kg) group. & Significantly different from limonoids (100 mg/kg) group.

## 5 Discussion

Aging is sometimes associated with cerebrovascular diseases that include neuro-inflammation which is referred to as “inflamm-aging”. This subclinical inflammation may lead to neurodegeneration and cognitive decline which represent feature of AD [29]. Accumulations of β-amyloid proteins, the appearance of neurofibrillary tangles, which are filaments formed of double helices and other proteins and neuronal degeneration, are the main histopathologic findings in AD. Also deficiency of acetylcholine "cholinergic hypothesis," is important in the development of AD symptoms. That is why enforcing the cholinergic function of the brain is the main target of AD therapy. Such therapy includes AChE inhibitors like donepezil, rivastigmine, and galantamine [1].

**Fig. 2:**
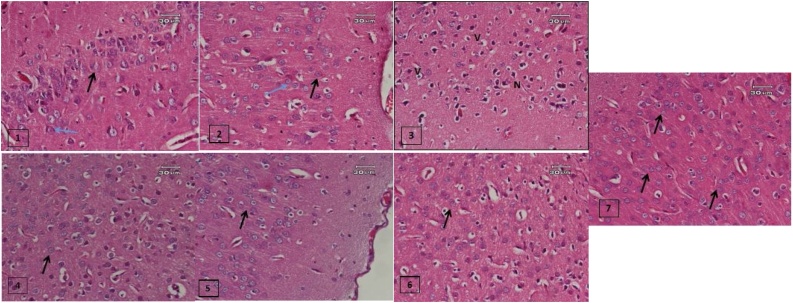
(1): A Photomicrograph of a section showing the normal control cerebral cortex of adult rats with the molecular layer containing nuclei of neuroglial cells, the granular layer containing numerous granular cells with rounded open face nuclei (black arrow) and small pyramidal cells with short apical dendrites (blue arrow) (H&E×400). (2): A Photomicrograph of a section of cerebral cortex of the donepezil (0.75 mg/kg) treated rats showing nearly normal neuronal cells (H&E×400). (3): A Photomicrograph of the cerebral cortex of the AlCl_3_ treated rats showing cellular vascular degeneration, atrophy, pyknosis, necrosis, neurology, congestion of cerebral blood vessels and focal cerebral haemorrhage (H&E×400). (4): Photomicrograph of the cortex of rat treated with low dose of limonoid showing marked improvement in most of the granule cells while the pyramidal are shown to be irregular in shape and surrounded by pericellular halos (H&E ×400). (5): A Photomicrograph of the cortex of rats treated with high dose of limonoid showing prominent regeneration where molecular layer contain nuclei of neuroglia cells and are covered with pia mater, and the granular layer contains numerous granular cells with rounded open face nuclei (H&E ×400).(6): A Photomicrograph of the cortex of rats treated with a low dose of hesperidin showing more or less improvement in granular cells and focal celluar pyknosis (H&E×400).(7): A Photomicrograph of the cortex of rats treated with a high dose of hesperidin showing nearly normal granular cells and neural structure (H&E ×400).

Aluminium is a trace element available in the Earth's crust naturally and has a toxic potential for humans. It has been suggested as a contributing factor in the pathogenesis of AD. Fadl et al. [23] stated that the administration of AlCl_3_ orally in a dose of 17 mg/kg body weight (b.w) daily for 45 days induced AD-like pathology in male rats with a significant increase in brain AChE activity [23]. In a former study done by Bihaqi et al. [24], they found that oral AlCl_3_ (50 mg/kg) elevated the enzymatic activity of AChE [24]. Also Auti and Kulkarni [25], in their study revealed that when AlCl_3_ (100 mg/kg) was administered orally daily for six weeks, it significantly increased cognitive dysfunction in rats [25].While Sharma et al. [26], reported that the administration of AlCl_3_ at a dose of 172 mg/kg/d orally for 10 weeks to rats caused brain oxidative stress [30]. That is why in the present study AlCl_3_ was given in four doses for four groups of rats, in order to select the most neurotoxic in the same duration of administration. In the current work, the dose of 172 mg/kg of AlCl_3_ had proven to be the most neurotoxic without any mortality in three weeks; that is why it had been used for induction of animal model with symptoms mimicking AD in humans, which was evaluated by comparing the effects of oral intake of AlCl_3_ by rats which represented the positive control group versus normal groups, on cognition as well as on psychological state of rats by doing behaviour stress tests. Also, biochemical parameters measured in brain homogenates and histopathologic examination of cerebral hemispheres of both groups confirmed the deleterious effects of AlCl_3_ on the brain tissue as shown in Fig. 2.

On the other hand, treatment with extracts of hesperidin and limonoids in the present study proved to be efficient in protecting against the deleterious effects of AlCl_3_ on the brain, as evidenced by increased percent of rat alternation in the Y - Maze test for rats receiving high and low doses of both extracts. The effects of both doses of hesperidin and a high dose of limonoids were nearly the same as that of the standard anti-cholinesterase drug; donepezil.

Regarding the efficacy of treatment on psychological activity tested by recording the ambulatory frequency in the open field, the effect of limonoids high dose was the best and better than the standard drug donepezil, and both doses of hesperidin, however the low dose of limonoids had no effect.

As for biochemical parameters: AChE, tau proteins and beta-amyloids, measured in brain homogenates; the effects of treatment with limonoids (50 mg/kg) were the best one followed by hesperidin (250 mg/kg), then hesperidin (125 mg/kg).The effect of treatment with extracts was better than the standard drug donepezil.These results showed that hesperidin effect was dose dependent, but the effect of limonoids was not dose dependent.

The evaluation of treatment results obtained from behaviour stress tests done using Y-Maze and Open field for the examination of cognition, ambulation abilities and exploration talents of rats were consistent with the results of biochemical parameters and histopathologic examination of the brains. All proved the efficacy of treatment with hesperidin in both doses and limonoids in high dose when compared to donepezil which is a standard drug that was used as a reference in the present study.

Flavonoids are known for their countless therapeutic activities [[31], [32], [33], [34], [35], [36]].The neuroprotective effect of hesperidin may be due to its nature as a flavanone glycoside, and being a phenolic compound that has the ability to cross the blood-brain barrier, rendered it as an inhibitor of progression of neurodegenerative diseases [37]. Parhiz et al. [38], revealed that the neuroprotective effects of hesperidin may be due to its mechanism of action via an ERK/Nrf2 signalling pathway which leads to its antioxidant cellular defences in addition to its radical scavenging activity being a phenolic compound. Also Roohbakhsh et al. [39] revealed that it possesses anti-inflammatory effects that contribute to its neuroprotective effect against brain aging. A previous study done by Francis et al. [40] and another one byShaaban et al. [41] referred to the ability of flavones to protect the brain cells due to their capability of increasing cerebral blood flow.

It is noteworthy that studies done by Visnagri et al. [42] and Ashafaq et al. [43], proved that the activities of AChE and Na^+^/K^+^ATPase (neurotoxicity markers) were markedly affected in diabetic animals receiving hesperidin. This finding enforces our results that showed an AChE lowering effect that contributed to its anti-AD activity based on the cholinergic hypothesis.

Hesperidin most probably interfered with the deposition of beta-amyloid in cerebral tissue in our study by its anti-inflammatory effect through down-regulation of transforming growth factor β1 as explained by Gray et al. [44], in previous studies who reported the role of growth factor β1in beta-amyloid production.

Limonoid glycosyl transferases are involved in carbohydrate acceptor molecules as well as in detoxification of biological toxins, xenobiotics, herbicides, pesticides and various polluting materials in plants [45]. In Human studies citrus limonoids and their derivates, have shown to possess good bioavailability [46], in addition to antioxidant and properties due to the presence of “limonoid glucoside that is the end product of limonoid glucosyl transferase (LGT) in citrus fruits [47], which may explain their neuroprotective effect in our study.

Combating neurodegenerative diseases as AD can be also achieved by metal chelation [48], Sun et al. [49] had proven in their study that limonoids possessed metal chelating properties thus could protect against AD. This can play a role in the explanation of the protective effect of a high dose of limonoids against AD.

## 6 Conclusion

Interest in the therapeutic role of fruit wastes increased dramatically over the last decade. Of particular interest are by-products that have an inhibitory effect on AChE and others that have antioxidative properties and can attenuate the toxicity induced by the β amyloid peptide. At this point, the aim of this study was to evaluate the anti-AD potential of *C. aurantium* by-products which is wasted in huge amounts. The effect of hesperidin and limonoid isolated from *C. aurantium* seeds in both doses were better than the standard drug donepezil and showed insignificant effects on AChE and β-amyloid levels when compared to normal control group. That is why our results encourage the use of *C. aurantium* seeds as AD preventive food additives especially for individuals at risk of developing age-related neurodegenerative diseases as those with the previous history of head trauma, strokes or at continuous risk of exposure to heavy metals as AlCl_3_ or lead or mercury.

## CRediT author statement

Doha H. Abou Baker: Principle investigator of the project no AR110214, chemistry and biochemistry part, idea, Writing and publishing of the manuscript. Bassant ibrahim: Pharmacology part, Nabilla hassan: Pathology part, Assmaa arafa: Language editing. Souad El Gengaihi: Reviewing and Editing.

## Declaration of Competing Interest

There is no Conflict of interest to declare.
